# Ruptured Tubercular Baker's Cyst: A Case Report

**DOI:** 10.1155/crrh/8840886

**Published:** 2025-03-26

**Authors:** Md. Rashid Al Mahmood, A. B. M. Mehedi

**Affiliations:** ^1^Physical Medicine and Rehabilitation, Bangladesh Medical College, Road 14/a, Dhanmondi, Dhaka 1209, Bangladesh; ^2^Physical Medicine and Rehabilitation, Ibn Sina Diagnostic Center, Jatrabari, Dhaka 1204, Bangladesh

**Keywords:** extrapulmonary TB, ruptured Baker's cyst, tubercular Baker's cyst

## Abstract

**Background:** Although tuberculosis (TB) can affect any organ, ruptured Baker's cyst due to TB is an uncommon phenomenon.

**Case Summary:** A young lady presented with unilateral atraumatic knee and calf pain and swelling. Pain characteristics shared both mechanical and inflammatory natures. Constitutional features for TB were evident. Based on history, clinical examination, and initial investigation, a ruptured Baker's cyst with septic knee was suspected. Ultrasound-guided aspiration followed by a synovial fluid study revealed inflammatory fluid with high adenosine deaminase level but without any bacterial growth in culture and negative GRAM and AFB staining. Empirical therapy was not curative. Commencement of anti-TB brought better clinicopathological outcome. Customized rehabilitation was set up.

**Conclusion:** Unilateral monoarthritis with ruptured tubercular Baker's cyst is a rare condition. Diagnosis, drug management, rehabilitation, follow-up, and recurrence prevention are challenging; especially with low resources. We took initiatives to collaborate all these.

## 1. Introduction

A Baker's cyst is a fluid-filled sac that forms in the popliteal fossa; hence, it is also called popliteal cyst. It typically involves the gastrocnemius-semimembranosus bursa and is located between the medial femoral condyle, semimembranosus tendon, and the medial head of the gastrocnemius. Baker's cyst is commonly associated with degenerative condition (osteoarthritis), inflammatory arthritis (rheumatoid arthritis and spondyloarthritis with peripheral involvement), infectious arthritis, pigmented villonodular synovitis, or traumatic meniscal tear. Sometimes it remains asymptomatic [1, 2]. Tuberculosis (TB) can virtually affect any and all organ systems. Skeletal TB accounts for about 10%–35% of cases of extrapulmonary TB and overall approximately 2% cases of TB. Baker's cyst infection with mycobacterium tuberculosis (MTB) is relatively rare [3, 4].

## 2. Case Description

Our patient, a 30-year-old women, Muslim, married, normotensive, nondiabetic, nonsmoker, housewife came with complaints of pain associated with swelling over left calf and knee for 1 month and difficulty in walking for 7 days. As per her statement, she was reasonably well 1 month back, then she developed left knee and calf pain which was gradually increasing, dull aching in nature, aggravated with both rest and activity, associated with swelling, night pain, and mild raised local temperature. She had low-grade evening rise of temperature occasionally for the same duration. Pain subsided initially with painkillers (cannot mention name) but gradually response was deteriorating. For last 7 days, the patient was unable to walk, difficulty was more on tiptoe stand or walk. She was unable to squat also.

She provided history of anorexia and weight loss (not documented), occasional photosensitivity but no history of other small or large joint pain, low back pain, morning stiffness, burning micturition, trauma, cough, respiratory distress, oral ulcer, rash, bleeding disorder, skin lesion, change in skin color, abnormal sensation, and contact with known TB patient.

She was immunized as per schedule. There was presence of Bacillus Calmette–Guerin (BCG) vaccine mark over her left arm.

She is blessed with one child of 5 year and gave history of two abortion and one stillbirth.

She belongs to lower middle-class family, reside in an apartment, and use low commode. All of her family members were enjoying good health.

She had no relevant past medical, hospitalization, or transfusion history.

She was free from tobacco or alcohol habits. She had no known drug reaction.

On general examination, the patient was ill looking, anemic, body build average, weight—53 kg, cooperative, normotensive (bp120/80), and afebrile. There was no lymphadenopathy, edema, or dehydration.

On musculoskeletal system examination, left calf was severely swollen with shiny surface, firm in consistency, reddish with increased temperature, and Grade 3 tender; the girth was around 5 cm more than the opposite side. No engorged vein was seen. Peripheral pulse was intact.

Left knee joint represented global swelling, reddish, mild raised temperature, Grade 3 tender, positive patellar tap, and enlarged tender Baker's cyst over the posteromedial aspect. Range of motion of examining knee was painful and limited.

The patient had weak planter flexor, and flexor hallucis longus (FHL) power was 3/5 on the affected side. Sciatic nerve stress tests were negative. Relevant neurological examination finding revealed no abnormality. Respiratory system and all other system were normal. Initial investigation finding revealed anemia with high ESR, high CRP, and neutrophilic leukocytosis. The Baker's cyst was present on imaging (Table 1).

### 2.1. Ultrasound-Guided Aspiration

**Figure 1:**
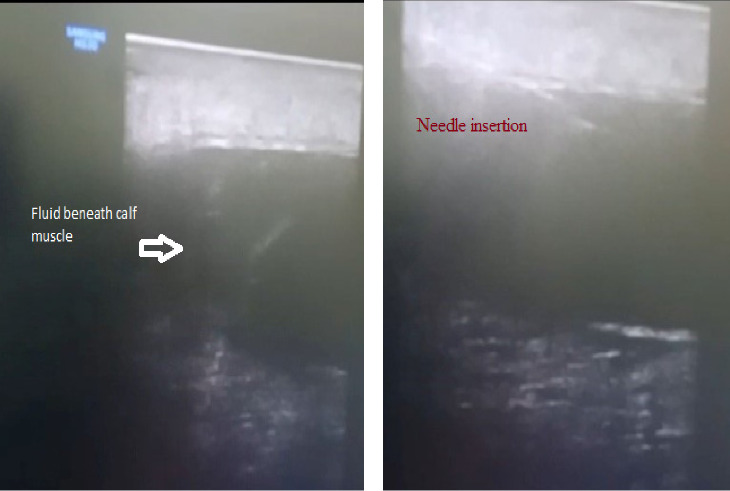
(a) Musculoskeletal ultrasound of the affected region showing large hypoechoic (with minimal hyperechoic) shadows resembling fluid (with debris) beneath muscle. (b) Ultrasound-guided needle insertion to aspirate fluid.

**Figure 2:**
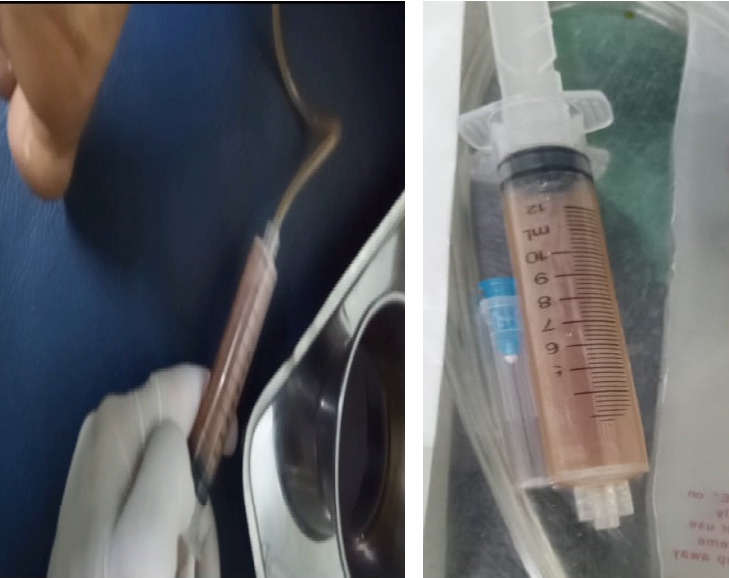
(a) Physician using syringe, attached with the transfusion set tube to aspirate fluid. (b) Disposable syringe containing the sample of aspirated fluid from Baker's cyst.

Initially, after proper explanation of the procedure and possible risks, consent was taken. Female attendance was ensured. Ultrasound screening was done by linear 12 hz transducer. Screening over the calf area revealed a large hypoechoic area in between the muscle (Figure 1(a)) that took the Doppler effect. Suprapatellar recess was also filled with fluid. Two physiatrists took part in the procedure. The whole area was sterilized with a disinfectant solution. Local anesthetic (2% lidocaine) was used over the skin of the port of entry. One transfusion set was taken, and tube part was cut and connected with a syringe while the other part contained wide bore needle. One examiner hold probe with left hand and positioned accordingly to ensure ultrasound-guided “in plane” needle insertion with the right hand (Figure 1(b)), while the other examiner waited for command and started aspiration with a syringe when requested (Figure 2(a)). There were multiple pockets on that area which needed multiple punctures to aspirate the whole fluid. Around 60 mL serosanguinous fluid was aspirated from the calf area (Figure 2(b)). After that, it was attempted from the knee joint. Around 20 mL similar fluid was aspirated from the medial and lateral approach. Procedure and postprocedure were uneventful. The immediate effect was subsidence of swelling and pain.

Fluid was sent for investigation and empirical therapy for septic knee and the ruptured Baker's cyst was commenced with 2 g intravenous ceftriaxone for 14 days, followed by oral cefixime 400 mg for 1 month and linezolid 400 mg for 6 weeks.  Table 2 represents the synovial fluid (SF) study.

### 2.2. Primary Follow-Up Findings

After 1.5 months, the patient reported clinical improvement of the knee and calf. Gait was nearly normal; planter flexor was 5/5 on the affected side. Calf and knee swelling mostly subsided and did not reappear. Despite these improvements, she still had persistent knee pain, limited range of motion, anorexia, weight loss (about 3 kg), anemia, and evening rise of temperature. Follow-up investigations are organized in Table 1. With the evidence of persistence constitutional feature (even after empirical management with antibiotic for 6 weeks), positive MT, high ADA in aspirated fluid, persistence high ESR, and availability of TB in Bangladesh, we decided to start anti-TB therapy (ATT) to the patient. We lacked gene expert of aspirated fluid and biopsy of synovium due to fund constraint. We tried to exclude other inflammatory joint conditions and relevant differential diagnosis by history, clinical examination, and necessary investigations.

### 2.3. Follow-Up Evidence After Anti-Tubercular Drug

We had plan to follow up first at the end of the initiation phase and then end of the sixth month, but the patient missed follow-up due to some unavoidable circumstances. She was under teleconsultation.

After 4 months, we had her follow-up (Table 1) that revealed anemia was corrected, and ESR CRP were normal. Clinically, she was free from fever, joint pain, reappearance of the Baker's cyst, weight loss, and anorexia. She had weight gain around 2 kg, had full ROM of affected knee, and was able to stand on her heel or tiptoe.

### 2.4. Rehabilitation Strategies

Exercise: Initially, relative rest was suggested for the affected joint. After acute stage subsidence, gradual pain-free range of motion was advised. Non–weight-bearing exercise were encouraged. Isometric quadriceps exercise was demonstrated and suggested.

Orthosis: During the acute stage, axillary crutch was suggested to keep the affected part free from weight bearing. As the knee cap may cause calf swelling, it was discouraged.

Activities of daily living (ADL) modification: The patient was suggested to use high commode, avoid sitting on the floor or stool which may need knee bending. She was advised to keep legs above the pillow while lying. To prevent friction with the opposite knee, supportive pillow was advised to take between the legs in case of lateral lying. Keeping the knee extended and resting over a stool while sitting were also mentioned. While bathing, a bath chair was suggested to use.

Physical modalities: Deep heating modalities, massage, or traction was not advised to use around the affected area. Warm moist compression and/or transcutaneous electrical neuromuscular stimulation (TENS) were options for physical therapy. Just immediate aspiration cold compression was advised for 2-3 days, 10 minutes in each setting.

## 3. Discussion

Bakers' cyst is situated on the posteromedial aspect of the knee. It may or may not communicate with the knee joint. If it ruptures, fluid may extend inferiorly along the gastrocnemius muscle into the calf or superiorly into the thigh along the semimembranosus. When Baker's cyst enlarges into the calf muscle, it can result in localized pain, swelling, erythema, or distal edema. It usually ruptures due to rapid accumulation of fluid or when pressure within the sac becomes too high. When the fluid is released into the surrounding tissues, it may result in sharp pain in the knee and calf, along with features of acute inflammation. Cyst rupture also may lead to additional complication including entrapment of the posterior tibial nerve, occlusion of the popliteal artery, foot drop, weakness of toes, and pain worsening with passive toe extension [1, 2]. Our patient presented with chronic insidious onset of symptoms with sudden deterioration of features, seemed as atraumatic ruptured Baker's cyst, communicated with the knee joint, and associated with complications.

Ultrasound-guided aspiration instead of surgical options can be performed to evaluate SF as there is lower recurrence rates in younger patient populations [1]. Also it is easier, patient friendly, and less hazardous. Ultrasound-guided aspiration is more specific and accurate than anatomical landmark–guided technique. So we attempted ultrasound-guided maneuver to aspirate the fluid. Both skilled interventionist and assistant are prerequisite for such procedure. The patient did not require further aspiration, which seems complete evacuation was probably done at the first attempt.

Usually, SF of septic arthritis reveals white blood cell (WBC) > 50,000/mm^3^ with > 90% polymorphonuclear neutrophil (PMN), while WBC 10000–50000/mm^3^ with > 50% PMN is commonly found in inflammatory arthritis [5]. The SF in joint TB demonstrates an inflammatory pattern with WBC counts over 10,000/mm^3^ (predominantly neutrophils), protein levels is elevated (higher than 3.5 g/dL), and joint fluid sugar level is usually low. However, SF cultures are positive in only 20%–40% of patients [6]. SF-ADA levels higher than 31 U*/*L were highly correlated with a diagnosis of TB arthritis, with high sensitivity (> 80%) and specificity (> 95%). SF-ADA may be considered as a less invasive and time-consuming diagnostic tool for TB arthritis [7]. Our case also had similarities with the above mentioned criteria for TB (inflammatory pattern neutrophilic leukocytosis, high protein, low sugar, and high ADA in SF).

All drug-sensitive TB (DS TB) patients, whether bacteriologically confirmed or clinically diagnosed, will receive the standard treatment regimen comprising of four drugs—isoniazid (5 mg/kg/day), rifampicin (10 mg/kg/day), ethambutol (15 mg/kg/day), and pyrazinamide (20 mg/kg/day)—for the initial 2 months (Intensive Phase) and two drugs—rifampicin and isoniazid—for the remaining 4 months (continuation phase). The continuation phase “may be” extended in case of joint TB to 10 months based on the clinical decision of the treating physician on a case-to-case basis [8]. Index Tb guideline from India and some other article also suggest 2 months intensive and 10–16 month continuation phase [9, 10].

We had initial follow-up evidence after the initiation phase and have plan to follow up to search for treatment failure, or relapse or any complications by clinicopathological markers. Healed status will be searched by completion of ATT and no relapse of disease for 2 years, along with resolution of fever, night sweats, weight loss, and no appearance of ulcer, sinus, or swelling.

Baker's cyst can occur primarily or secondarily. It is in direct communication with the knee joint and acts as a one-way valve, allowing the fluid to flow into the cyst but not the other way around. Ruptured Baker's cyst closely resembles deep vein thrombosis, which may delay the diagnosis. It can present with swelling and redness in the calf region. A number of knee diseases, including chondral lesions, inflammatory arthritis, osteoarthritis, anterior cruciate ligament tears, meniscus tears, and effusion, have been connected to this condition. The most frequently associated arthropathy with Baker's cyst is osteoarthritis, followed by inflammatory arthritis, whereas infective etiology is rare, and tubercular origin is exceptional. Several studies of Baker's cyst supported the rare occurrence of tubercular pathology [11–14]. But in an endemic area such as Bangladesh, TB can be presented in different forms, so a differential diagnosis should be kept in mind.

## 4. Conclusion

Ruptured Baker's cyst due to TB is a disease of exclusion as its not much a familial reason for the Baker's cyst.Ultrasound-guided aspiration helps to evacuate all accumulated fluid which lessen patient's treatment hazard. Initially although her synovial ADA was suggestive, but AFB stain and culture negative reports were one of the reasons to delay start anti-TB drugs. Despite empirical antibiotics for the 6-week patient had partial improvement with persistence common constitutional features for TB, for which we reconsidered starting anti-TB drugs. The patient improved markedly after ATT. Customized rehabilitation strategies were shared with the patient.

### 4.1. Limitation

We lacked gene expert and synovial biopsy; any or both would become strong evidence. Due to some unavoidable circumstance, initial follow-up after ATT was delayed by the patient.

## Figures and Tables

**Table 1:** Investigation finding (initial, after empirical therapy, and after anti-TB therapy).

Investigation	Initial findings	Follow-up after empirical therapy	Follow-up after antitubercular therapy
Hb (g/dL)	9.9	11.6	12.5
Erythrocyte sedimentation rate (ESR) (at 1st hour)	82	58	05
Wbc (per mm^3^/cmm)	15,500	8000	8800
CRP (mg/L)	39	5	0.85
Sgpt (U/L)		25	20
S creatinine (mg/dL)	0.93		0.96
Uric acid (mg/L)	5.89		
Rheumatoid factor	Negative		
Duplex study of the affected lower limb	Normal arterial and venous system Baker's cyst present		
Montaux test (MT)		Positive (14 mm)	
X-ray sacroiliac joint–modified Ferguson view		Osteitis condensus illi (bilateral)	
ANA		Negative	
ANTI DS DNA		Negative	
RBS (mmol/L)		6	
X-ray knee standing both view		Normal	

**Table 2:** Aspirated fluid study.

Color	Reddish
WBC (per mm^3^/cmm)	43,000
Polymorph	95%
Glucose (mmol/l)	< 0.03 (low)
Protein (g/L)	50.27 (high)
ADA (u/l)	68.25 (increased)
GRAM stain and acid fast bacilli (AFB stain)	Negative
Culture	No growth

## Data Availability

Data are available on reasonable requests only.

## Nomenclature

TB: Tuberculosis
ESR: Erythrocyte sedimentation rate
CRP: C-reactive protein
ADL: Activities of daily living
BCG: Bacillus Calmette–Guerin
FHL: Flexor hallucis longus
ATT: Anti-TB therapy
MTB: Mycobacterium tuberculosis
SF: Synovial fluid
